# Nomogram Based on Systemic Immune-Inflammation Index to Predict Survival of Tongue Cancer Patients Who Underwent Cervical Dissection

**DOI:** 10.3389/fonc.2020.00341

**Published:** 2020-03-11

**Authors:** Zhiyuan Lu, Wangxiang Yan, Jianfeng Liang, Mei Yu, Jiayu Liu, Jiansuo Hao, Quan Wan, Jiameng Liu, Chongdai Luo, Yiyang Chen

**Affiliations:** ^1^Department of Oral and Maxillofacial Surgery, Stomatology Medical Center, Guangzhou Women and Children's Medical Center, Guangzhou, China; ^2^Department of Oral and Maxillofacial Surgery, The First Affiliated Hospital, Sun Yat-Sen University, Guangzhou, China; ^3^Department of Oral and Maxillofacial Surgery, Hospital of Stomatology, Sun Yat-Sen University, Guangzhou, China

**Keywords:** tongue squamous carcinoma cancer, systemic immune-inflammation index, overall survival, nomogram, prognostic prediction

## Abstract

**Aim:** The aim of this study was to evaluate the prognostic significance of the preoperative systemic immune-inflammation index (SII) and to establish a nomogram for prediction of survival of tongue squamous cell carcinoma (TSCC) patients who underwent primary surgery and cervical dissection.

**Methods:** 120 patients diagnosed with TSCC who underwent primary tumor and neck dissection without preoperative treatment were included to develop the nomogram. This model was externally validated in an independent data cohort of 50 TSCC patients. X-tile software was used to identify the optimal cut-off value. Prognostic factors were identified by Univariate and multivariate analyses. A nomogram based on the multivariate analysis results was built to predict the survival rate and calibration curves and concordance index (C-index) were used to determine predictive and discriminatory capacity.

**Results:** The optimal cut-off value was 569×10^9^/L for SII. In the training cohort, a high preoperative SII (>569) was significantly related to tumor size, histological grade, depth of invasion, lymph node density (LND). A Kaplan-Meier survival analysis showed that patients with a lower SII had a significantly better 5-year overall survival (OS) and disease-free survival (DFS) than patients with high SII (80.8% vs. 43.5% and 72.7% vs. 36.2%, respectively, P<0.001). Univariate analyses of training cohort revealed that age, clinical stage, depth of invasion, LND, neutrophil-to-lymphocyte ratio (NLR), platelet-to-lymphocyte ratio (PLR) and SII were significant prognostic factors for OS. Moreover, the receiver operating characteristics (ROC) curve showed that SII was superior to NLR and PLR for predicting clinical outcomes. However, multivariate analysis found that age, LND, and SII were independent risk factors for OS. The C-index of the nomograms based on independent prognostic factors was 0.716 for OS and 0.723 for DFS. The C-indexes for external validation of OS and DFS were 0.852 and 0.754, respectively. The calibration curves showed good agreement between predicted and actual observations of OS and DFS.

**Conclusion:** SII can serve as a novel independent prognostic factor for OS and DFS of patients with TSCC. The prognostic nomogram based on SII is a reliable model for predicting survival of patients with TSCC after surgery.

## Introduction

Oral cancer is one of the most common malignant tumors in the head and neck region, and incidence rates are rising around the world (1, 2). Tongue squamous cell carcinoma (TSCC) is one of the most aggressive tumors of oral cancers, characterized by high incidence of cervical lymph node metastasis (3). Currently, surgery remains the main treatment choice for localized TSCC, but the prognosis of patients remains unsatisfactory; in some studies, ~40–60% patients suffer from local recurrence or lymph node metastasis within 5 years, even after curative treatment (4).

At present, the prognosis and treatment of TSCC patients is primarily determined by the tumor node metastasis (TNM) staging classification (5). However, the prognosis of patients with TSCC with the same TNM stage is highly variable since it is influenced by a variety of factors (6). Recent reports have suggested that many molecular biomarkers involved in angiogenesis, metastasis, proliferation, and differentiation could be used to improve prognostic precision for TSCC patients (7–9). However, these expensive laboratory techniques and comprehensive tests are rarely suitable for TSCC patients. Identifying novel prognostic parameters obtained before surgery may provide useful insights to help clinicians choose more suitable treatments for TSCC patients.

Increasing evidence has confirmed that systemic inflammatory responses and the immune system play important roles in the tumor microenvironment (10–12). Several peripheral indicators of immunity/inflammation, including the neutrophil-to-lymphocyte ratio (NLR), platelet-to-lymphocyte ratio (PLR), and lymphocyte to monocyte ratio (LMR) are used as significant prognostic indicators in many solid tumors, including TSCC (13, 14). Recently, the systemic immune-inflammation index (SII), based on a combination of peripheral lymphocytes, neutrophils, and platelet count, was hypothesized to better reflect the balance between host inflammation and immune status. Its prognostic value in hepatocellular carcinoma (15), gastric cancer (16), colorectal cancer (17), and small cell lung cancer (18) has been confirmed, but few studies have focused on the importance of SII in TSCC.

Nomograms are convenient and advanced methods that use two or more known variables to estimate clinical events, and are currently widely used for prognostic prediction in most cancer types (19–21). This study was designed to determine whether SII, when combined with other prognostic and clinicopathological factors, can create a nomogram to conveniently estimate the 3- and 5-year overall survival (OS) and disease-free survival (DFS) for resectable TSCC patients.

## Patients and Materials

### Patients and Data Collection

A total of 120 patients with pathologically proven TSCC, diagnosed at the First Affiliated Hospital of Sun Yat-sen University between March 2012 and November 2017, were retrospectively analyzed. All patients received primary surgical resection and cervical dissection but had not undergone preoperative cancer treatment. Patients who had recurrent tumors were excluded from this study. Patients with inflammatory or autoimmune diseases were also excluded. An independent cohort of patients with TSCC who met the same eligibility criteria in Hospital of Stomatology of Sun Yat-sen University from January 2013 to December 2017 was collected as the external validation cohort for this study. Tumor stages were classified according to the 7th edition Cancer Staging of American Joint Committee on Cancer. Post-operative radiotherapy or concurrent chemoradiotherapy was performed if patients with pathologically-diagnosed as positive lymph nodes or pathologic T3/T4 tumors, with the exception of patients who refused treatment.

Peripheral blood samples of the patients were collected within 1 week before surgery. The SII was defined as follows: NLR = neutrophil count/lymphocyte count; PLR = platelet count/lymphocyte count; LMR = lymphocyte count/monocyte count; SII = platelet count × neutrophil count/lymphocyte count. The optimal cut-off values for the above indexes were obtained using X-tile software (https://x-tile.software.informer.com/). All blood cell assessments were performed in our institutional laboratory according to standard operating procedures. The clinical and pathological data collected included age at diagnosis, gender, Eastern Cooperative Oncology Group-Performance Status (ECOG-PS), histologic grade, depth of invasion, treatment modality, and lymph node involvement.

In survival analysis, the overall survival (OS) was calculated from the date of surgery to the date of death or last follow-up. The disease-free survival (DFS) time was calculated from the operation date to the date of recurrence, death, or last follow-up. The last follow-up was November 2019 for both training cohort and validation cohort. Written consent was obtained from all enrolled patients and the study was approved by the Institutional Review Board of the First affiliated Hospital and Hospital of Stomatology of Sun Yat-sen University.

### Statistical Analysis

The relationships between SII and other key clinicopathological characteristics were analyzed by Chi-square test or Fisher's exact test. Prognostic factors for OS and DFS were assessed by univariate and multivariate Cox proportional hazards models. The Kaplan-Meier method and log-rank test were used to compare the survival of different groups. Statistical analyses were performed using the SPSS Statistic software 22 package (SPSS Inc., Chicago, III), and *P* < 0.05 was considered statistically significant. Receiver operating characteristics (ROC) curves and area under the ROC curve (AUC) were used to compare prognostic factors.

The nomogram was formulated using the R software “rms” package (Version 5.1–0, Vanderbilt University, Nashville, TN) with endpoints of 3-year and 5-year OS and DFS. The concordance index (C-index) was calculated to determine the accuracy of the nomogram in predicting OS and DFS. The calibration plots of nomograms were used to assess the consistency between the predicted survival and the observed survival.

## Results

### Clinical Characteristics of Patients

The training cohort included 120 TSCC patients treated with resection of the primary tumor site and cervical. The validation cohort consisted of 50 patients. All patients' clinicopathologic characteristics are described in Table 1. In the training cohort, the median age at diagnosis was 55 (range 22–86) years. Of them, 79 (65.8%) were male and 41 (34.2%) were female. There were 29 patients (24.2%) at early stages (I and II) and 91 (75.8%) patients at late stages (III and IV). There were 65 cases of poor-moderate differentiated tumors and 55 cases of well differentiated tumors. There were 40 patients underwent postoperative adjuvant radiotherapy, while 12 patients received postoperative chemoradiotherapy in training cohort group. A total of 53.3% patients (*n* = 64) had lymph node metastasis and 46.7% patients (*n* = 56) had no metastasis. The mean depth of invasion was 7.3 (range, 1–35) mm in training group and 8.5 (range, 2–33) mm in validation group. The mean values of peripheral lymphocytes, neutrophils, platelets, and monocytes were 2.11, 4.04, 238, and 0.57 × 10^9^ cells/L in the training cohort, respectively.

**Table 1:** Clinicopathological characteristics of patients with TSCC.

	**Training cohort (*****n*** **=** **120)**		**Validation cohort (*****n*** **=** **50)**	
**Variables**	***n***	**%**	***n***	**%**
**Age**				
<55	58	48.3	28	56
≥55	62	51.7	22	44
**Gender**				
Male	79	65.8	30	60
Female	41	34.2	20	40
**ECOG PS**				
0	87	72.5	46	92
1	33	27.5	4	8
**Tumor size (T)**				
T1-T2	69	57.5	32	64
T3-T4	51	42.5	18	36
**Nodal metastasis**				
N0	56	46.7	28	56
N1	27	22.5	13	26
N2	34	28.3	9	18
N3	3	2.5	0	0
**TNM stage (AJCC, 7TH)**				
I-II	29	24.2	22	44
III-IV	91	75.8	28	56
**Histological grade**				
Well	55	45.8	27	54
Poorly/moderately	65	54.2	23	46
**Depth of Invasion (MM)**				
Mean (range)	7.3 (1–35)		8.5 (2–33)	
**Treatment**				
Surgery only	68	56.7	33	66
Surgery + radiotherapy	40	33.3	13	26
Surgery + chemoradiotherapy	12	10	4	8
**Neutrophil (×10**^**9**^**/L)**				
Mean (range)	4.04 (1.46–10.02)		4.20 (1.00–15.67)	
**Lymphocyte (×10**^**9**^**/L)**				
Mean (range)	2.11 (0.70–3.73)		1.85 (0.68–3.30)	
**Platelet (×10**^**9**^**/L)**				
Mean (range)	238 (74–513)		218.34 (135–379)	
**Monocyte (×10**^**9**^**/L)**				
Mean (range)	0.57 (0.23–1.63)		0.633 (0.21–1.40)	

*ECOG PS, Eastern Cooperative Oncology Group performance status*.

### Relationship Between Clinical Characteristics and SII in the Training Cohort

The optimal cut-off value of SII for predicting survival was determined to be 569 × 10^9^/L by X-tile software (Supplemental Figure S1). The optimal cut-off values of NLR, PLR, and LMR were also determined. The correlation between SII and clinicopathological characteristics is shown in Table 2. Lymph node density (LND) was a reliable prognosis factor, and was calculated by number of positive lymph nodes/ total number of lymph nodes. The optimum cut-off value for LND determined by X-tile software was 0.057 (Supplemental Figure S1). High SII was been found to be associated with histological differentiation (*P* = 0.011), tumor size (*P* = 0.002), depth of invasion (*P* = 0.011), and LND (*P* = 0.003) in the training cohort.

**Table 2:** Relationship between baseline characteristics and SII.

**Variable**	**SII ≤ 569 (*n* = 82)**	**SII > 569 (*n* = 38)**	**χ^2^**	***P***
**Age**				
<55	43	15	1.748	0.186
≥55	39	23		
**Gender**				
Male	54	25	<0.001	0.994
Female	28	13		
**Tumor size (T)**				
T1-T2	55	14	9.711	0.002*
T3-T4	27	24		
**Lymph node metastasis**				
N0	43	13	3.467	0.063
N+	39	25		
**TNM staging**				
I-II	23	6	2.129	0.144
III-IV	59	32		
**Histological grade**				
Well	44	11	6.387	0.011*
Poorly/moderately	38	27		
**LND**				
LND ≤ 0.057	58	16	9.002	0.003*
LND > 0.057	24	22		
**Depth of Invasion**				
≤5 mm	52	13	9.000	0.011*
5 <DOI≤10 mm	23	19		
>10 mm	7	6		

DOI, depth of invasion; LND, lymph node density; SII, immune-inflammation index; *P value < 0.05.

### High SII Is Associated With a Poor Prognosis in the Training Cohort

**Figure 1:**
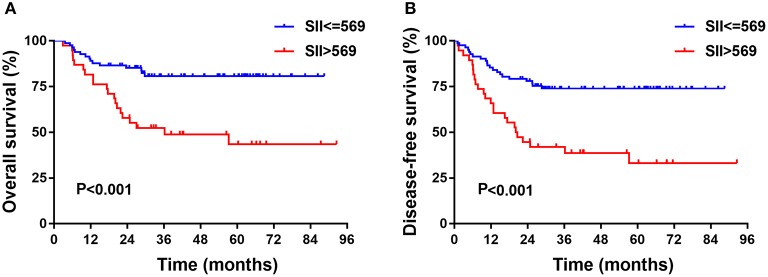
Kaplan-Meier analysis of OS **(A)** and DFS **(B)** or SII of patients after radical operation for TSCC in training cohort. OS, overall survival; DFS, disease-free survival; SII, immune-inflammation index; TSCC, Tongue squamous cell carcinoma.

In the training cohort, the median follow-up time was 37.5 months (range, 3–92). There were 35 patients who died and 18 patients who experienced disease recurrence at last follow-up. The 5-year OS and DFS of the training cohort were 68.7 and 60.8%, respectively. Univariate analysis revealed that age, clinical stage, depth of invasion, LND, NLR, PLR, and SII were prognostic indicators of OS. Univariate analysis revealed that age, histological grade, clinical stage, depth of invasion, LND, NLR, PLR, and SII each had statistically significant associations with DFS. Kaplan-Meier analyses revealed that patients with high SII had worse prognosis, including OS and DFS, compared with the low SII group (*P* < 0.001, HR: 3.395, 95% CI: 1.736–6.640 and *P* < 0.001, HR: 2.825, 95% CI: 1.572–5.077, respectively, Figure 1). Of note, multivariate analysis indicated that, among the inflammation indexes, only SII was an independent prognostic parameter for OS and DFS in patients with resectable TSCC (Table 3).

**Table 3:** Univariate and multivariate analysis of variables associated with overall and disease free survival in training cohort.

**Variable**	**OS**				**DFS**			
	**Univariate analysis**		**Multivariate analysis**		**Univariate analysis**		**Multivariate analysis**	
	**HR 95% CI**	***P***	**HR 95% CI**	***P***	**HR 95% CI**	***P***	**HR 95% CI**	***P***
**Age**								
<55	Reference		Reference		Reference		Reference	
≥55	2.017 (1.003–4.005)	0.049^*^	2.148 (1.063–4.342)	0.033^*^	2.472 (1.314–4.650)	0.005^*^	3.332 (1.730–6.414)	<0.001^*^
**Gender**								
Male	Reference		–		Reference		–	
Female	0.961 (0.478–1.931)	0.910	–		0.807 (0.429–1.517)	0.506	–	
**Tumor size (T)**								
T1-T2	Reference		–		Reference		–	
T3-T4	1.678 (0.863–3.265)	0.127	–		1.504 (0.838–2.700)	0.172	–	
**LND**								
LND ≤ 0.057	Reference		Reference		Reference		Reference	
LND > 0.057	3.011 (1.527–5.938)	0.001^*^	2.463 (1.207–5.027)	0.013^*^	2.555 (1.416–4.610)	0.002^*^	2.029 (1.066–3.861)	0.031^*^
**TNM staging**								
I–II	Reference		–		Reference		–	
III–IV	2.953 (1.041–8.377)	0.042^*^	–	0.257	2.518 (1.065–5.956)	0.035^*^	–	0.536
**Differentiation**								
Well	Reference		–		Reference		Reference	
Poorly/moderately	1.821 (0.905–3.664)	0.093	–		2.130 (1.144–3.966)	0.017^*^	2.266 (1.164–4.412)	0.016^*^
**DOI**								
≤5 mm	Reference				Reference			
5 <DOI≤10 mm	2.289 (1.082–4.843)	0.030^*^	–	0.249	1.621 (0.850–3.092)	0.140	–	0.692
>10 mm	3.362 (1.320–8.566)	0.011^*^	–	0.423	2.593 (1.134–5.933)	0.024^*^	–	0.501
**NLR**								
NLR ≤ 2.8	Reference		–		Reference		–	
NLR >2.8	3.264 (1.565–6.807)	0.002^*^	–	0.165	2.417 (1.195–4.891)	0.014^*^	–	0.595
**PLR**								
PLR ≤ 140.5	Reference		–		Reference		–	
PLR > 140.5	2.652 (1.356–5.185)	0.004^*^	–	0.120	2.324 (1.271–4.250)	0.006^*^	–	0.194
**LMR**								
LMR ≤ 4.02	Reference		–		Reference		–	
LMR > 4.02	0.931 (0.478–1.811)	0.832	–		0.733 (0.405–1.324)	0.303	–	
**SII**								
SII ≤ 569	Reference		Reference		Reference		Reference	
SII > 569	3.395 (1.736–6.640)	<0.001^*^	2.613 (1.295–5.275)	0.007^*^	2.825 (1.572–5.077)	<0.001^*^	1.439 (1.012–3.715)	0.046^*^

*OS, overall survival; DFS, disease-free survival; CI, confidence interval; HR, hazard ratio; LND, lymph node density; DOI, depth of invasion; NLR, neutrophil to lymphocyte ratio; PLR, platelet to lymphocyte ratio; LMR, lymphocyte to monocyte; SII, immune-inflammation index*.

* *P value < 0.05*.

**Figure 2:**
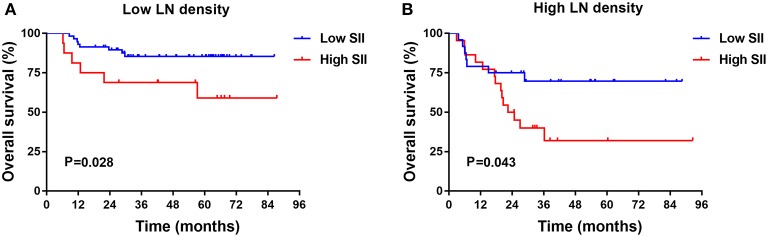
Kaplan-Meier survival analysis in low **(A)** and high **(B)** lymph node (LN) metastasis rate TSCC subgroups in training cohort.

The results indicated that LND was a significant prognostic factor for DFS and OS by univariate and multivariate analysis. Patients with low SII had longer OS than those with high SII in the low LND group (*P* = 0.028, Figure 2A). In the high LND group, we found that patients with low SII had significantly better OS compared with patients high SII (*P* = 0.043, Figure 2B).

**Figure 3:**
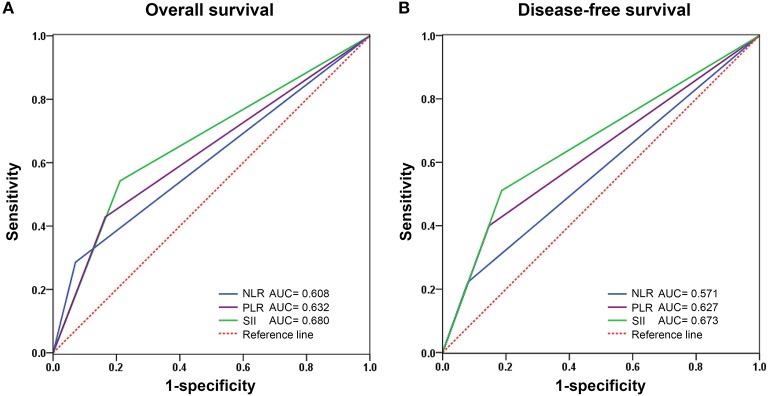
Predictive ability of SII compared with NLR and PLR for overall **(A)** and disease-free survival **(B)** by receiver operating characteristic (ROC) curve analysis in training cohort. NLR, Neutrophil-lymphocyte ratio; PLR, Platelet-lymphocyte ratio; SII, Systemic immune-inflammation index.

The prognostic value of the NLR, PLR, and SII index was evaluated by comparing the AUC area. The AUC of the NLR, PLR, and SII for OS were 0.608, 0.632, and 0.680, respectively (Figure 3A), and the AUC for DFS were 0.571, 0.609, and 0.656, respectively (Figure 3B), indicating that SII is superior to other inflammatory indexes.

### Predictive Accuracy of Nomogram for OS and DFS

**Figure 4:**
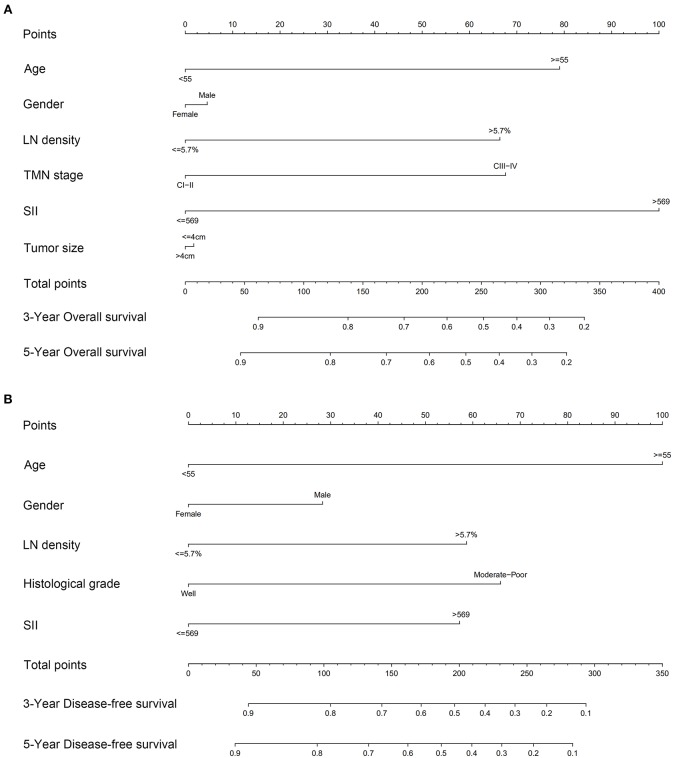
Nomograms to predict 3- and 5-year **(A)** overall survival and **(B)** disease-free survival for patients with TSCC.

Based on the multivariate analysis results, independent risk factors were integrated into nomograms to predict the 3- and 5-year OS. These risk factors included age, LND, and SII (Figure 4A). Regarding prediction of DFS, only age, histological differentiation, SII, and LND were independent prognostic factors (Figure 4B).

**Figure 5:**
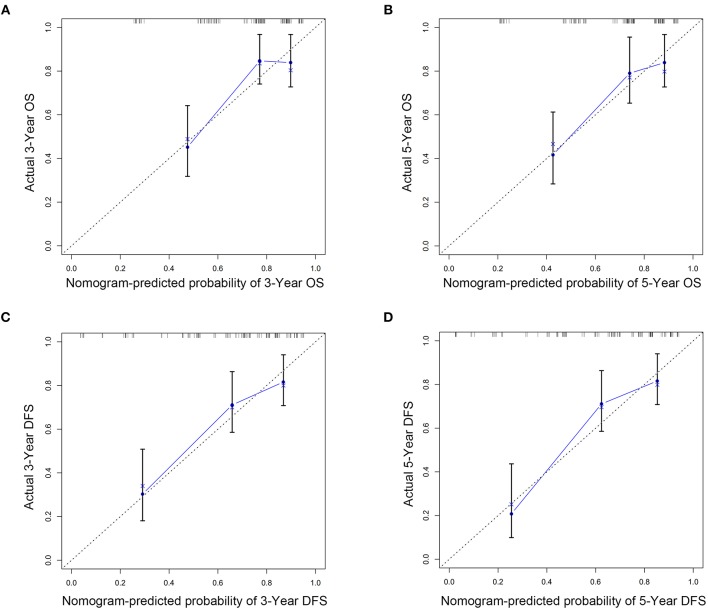
Nomogram model calibration curves of 3-year **(A)** and 5-year **(B)** overall survival and 3-year **(C)** and 5-year **(D)** disease-free survival in training cohort.

**Figure 6:**
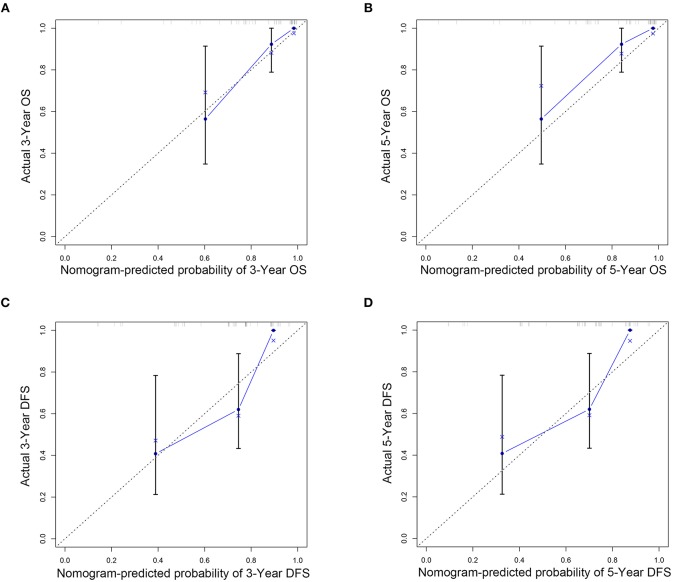
Nomogram model calibration curves of 3-year **(A)** and 5-year **(B)** overall survival and 3-year **(C)** and 5-year **(D)** disease-free survival in external validation cohort.

**Figure 7:**
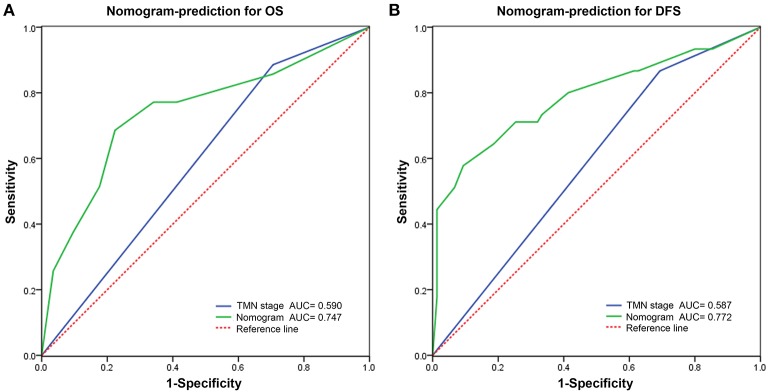
Predictive performance of nomogram compared with TNM stage for overall survival **(A)** and disease-free survival **(B)** by receiver operating characteristic (ROC) curve analysis.

The C indexes of the nomogram were 0.716 (95%CI: 0.624–0.808) for OS and 0.723 (95%CI: 0.643–0.803) for DFS, demonstrating good predictive accuracy for survival of TSCC patients after surgery. The external validation cohort was used to test the predictive value of the nomogram. The C-index of the nomogram was 0.852 (95%CI, 0.764–0.940) for OS and 0.754 (0.652, 0.856) for DFS. The calibration curve showed that the 3- and 5-year OS and DFS predicted by the nomogram were consistent with actual observations (Figures 5, 6). Moreover, we compared the predictive accuracies for OS and DFS between the nomogram and the TNM staging system. The AUC of the nomogram was significantly higher than the 7th TNM staging system in OS (0.747 vs. 0.590) and DFS (0.772 vs. 0.587) (Figure 7). The above results indicate that the nomogram incorporating SII and LND had better performance in predicting OS and DFS of patients with TSCC than AJCC 7th staging system.

## Discussion

In this study, we provide evidence that a high level of SII may be an independent significant risk factor in patients with TSCC. To our knowledge, this is the first study to establish and externally validate a nomogram model for OS and DFS that incorporates SII markers and clinicopathological characteristics to improve predictive accuracy for TSCC patients.

Currently, TNM staging is the most common prognostic tool for determining TSCC prognosis. However, the TNM staging system has a few limitations for survival analysis (22, 23). Importantly, it focuses only on tumor characteristics without accounting for other factors that affect prognosis, such as inflammatory biomarkers. For this reason, we sought to identify clinically significant and inexpensive-to-measure prognostic factors that were available at the time of diagnosis. Increasing research has indicated a significant link between characteristics of the systemic inflammatory response and various types of cancers (24). To date, many studies have shown that several pretreatment inflammatory indexes such as NLR, LMR, and PLR are significant factors in the progression and prognosis of different cancer types; these indexes can each be used as a single prognostic indicator or can be combined (25). SII is a relatively new index that reflects inflammation status and is correlated with circulating tumor cells. A high level of SII has been associated with advanced clinicopathological characteristics and has been identified as a reliable prognostic factor for long term survival in various malignant tumors (26). SII has also been proposed as a better reflection of inflammatory status and prognosis than other inflammatory markers in many cancers (27–29). A study conducted by Jomrich et al. (30) revealed that SII was a better prognostic factor than NLR and PLR by ROC analysis in pancreatic ductal adenocarcinoma patients who underwent resection.

However, the prognostic implications of SII on TSCC have not been studied to date. To our knowledge, this is the first report to investigate the prognostic value of SII in TSCC patients who underwent primary site and cervical dissection. In this work, we established a nomogram based on independent prognostic factors to predict long-term survival in TSCC patients. We retrospectively calculated SII from the blood cell count of 120 patients and identified an optimal cut-off value of 569 × 10^9^/L using X-tile software. The correlations between SII and clinicopathological characteristics were analyzed, and our results showed that high SII was related to tumor size, histological grade, depth of invasion, and LND in patients with TSCC. LND has recently been reported as one of the most valuable prognostic indicators for outcomes in head and neck cancers (31). A previous study showed that LND was a more reliable predictor than pathologic node stage in oral cancer (32). In this work, we found that a high LND ratio (>0.057) was associated with worse OS and DFS for patients with TSCC, in good agreement with prior studies (33). We studied the association between SII and LND and found that, regardless of LND categorization (low or high), patients with low SII tended to survive longer than those with higher SII. In addition, univariate analysis results showed that LND, SII, age, clinical stage, depth of invasion, NLR, and PLR were significantly associated with OS. This finding was consistent with the results of a previous study by Diao et al. (34) that reported that high preoperative SII with a cut-off value of 484.5 was associated with poor OS in oral cancer. SII was also superior to NLR and PLR as an indicator for OS and DFS as determined by ROC analysis, similar to a previous study of gastric cancer (16). Taken together, these data suggest that SII may be a superior index for survival prediction of TSCC.

Although the prognostic value of these inflammatory biomarkers in cancers seems clear, the mechanisms by which they contribute to improved survival outcomes require further detailed study. Lymphocytes are an important part of the immune response and destroy residual cancer cells by recognizing tumor antigens (35). Low lymphocyte counts have been reported to accelerate the development and progression of tumors (36). Neutrophils are capable of secreting chemokines and cytokines which may create a favorable environment for tumor growth by remodeling the extracellular matrix and angiogenesis (37, 38). A high number of intratumoral neutrophils has been linked to worse prognosis in multiple cancers (39). Thus, a higher SII indicates an imbalance of the inflammatory response, which might be linked to tumor invasion and worse prognosis.

Nomograms have become a reliable predictive tool and are widely applied in clinical use. Currently, some nomograms have been reported to predict clinical outcomes in oral cancer (40, 41), but few studies have been published based on SII as a risk factor. The multivariate analysis revealed that several clinicopathological characteristic were independent negative predictors of OS, including age, LND, and SII. We successfully established nomograms based on significant independent factors that accurately predicted the 3- and 5-year survival rates of patients with TSCC. The results of the C-index, AUC, and calibration curve from both the training and validation cohorts showed the reliable discriminative performance and predictive accuracy of these nomograms. Moreover, the nomograms in combination with several other clinicopathological parameters appeared to improve predictive ability compare with 7th TNM staging system. The nomograms have the potential to be a reliable model for predicting survival in postoperative TSCC patients, but it still requires more research.

This study had several limitations. This was a retrospective single-center study with a small cohort, which have selection bias. The extranodal extension was considered to be an independent factor associated with survival in TSCC patients after surgical resection. However, our study included patients from 2012 to 2017. In the early years, there were too many deletions for the extranodal extension. Thus, extranodal extension was not included in our research. The cut-of values of SII identified by this institution were inconsistent with results of previous research, which requires larger sample size from subsequent prospective studies to validate the result. Despite these limitations, our clinical data suggest that the serum SII index is a convenient and noninvasive method that could serve as a strong predictor of OS in TSCC patients.

In conclusion, pre-treatment SII was a useful prognostic factor for OS and DFS in TSCC patients. A nomogram based on SII was helpful in improving the accuracy of clinical prognoses for TSCC patients undergoing primary site and cervical dissection. Considering the convenience of this measure, peripheral SII has great potential as a candidate for further research and clinical application.

## Data Availability Statement

The datasets generated for this study are available on request to the corresponding author.

## Ethics Statement

Written consent was obtained from all enrolled patients and the study was approved by the Institutional Review Board of the First affiliated Hospital of Sun Yat-sen University and Hospital of Stomatology of Sun Yat-sen University.

## Author Contributions

YC conceived of, designed, and supervised the study. WY, JiayL, JH, and QW collected the data of patients and followed up. WY, ZL, JiayL, and CL analyzed the data. MY and JianL provided technical assistance with the data analysis. YC, WY, and ZL wrote the manuscript. All co-authors have reviewed and approved this version of the manuscript.

### Conflict of Interest

The authors declare that the research was conducted in the absence of any commercial or financial relationships that could be construed as a potential conflict of interest.

## Supplementary Material

Supplementary Figure S1 Identification of optimal cut-off values of SII **(A)**, LND, NLR, PLR, and LMR **(B,C,D,E)** via X-tile analysis.
