# Supplementary figures and images for: Nomogram Based on Systemic Immune-Inflammation Index to Predict Survival of Tongue Cancer Patients Who Underwent Cervical Dissection
Source: Front Oncol. 2020 Mar 11;10:341. doi: 10.3389/fonc.2020.00341 (PMC7078378; doi:10.3389/fonc.2020.00341)

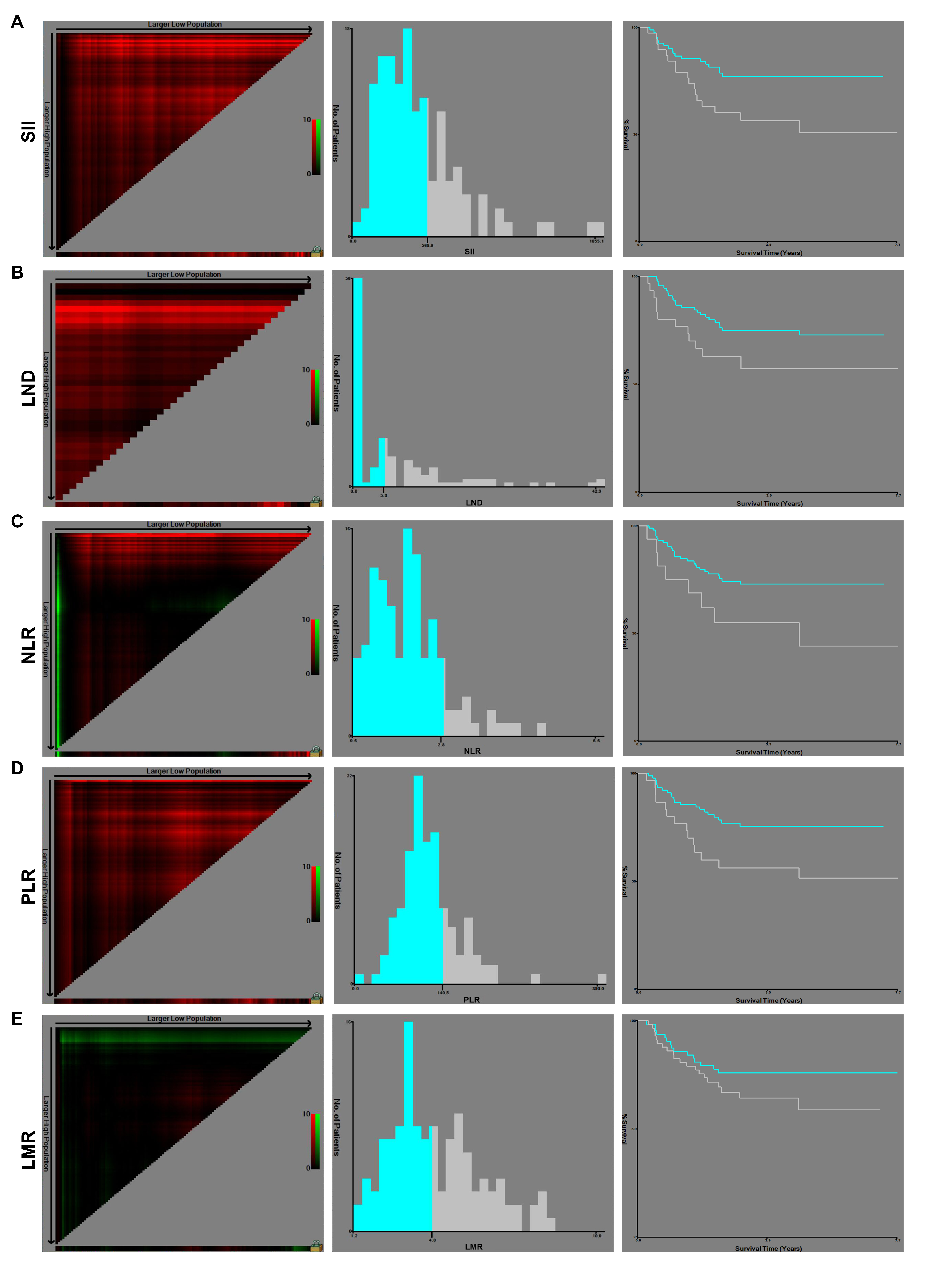

Supplement: Supplementary Figure S1 — Identification of optimal cut-off values of SII (A), LND, NLR, PLR, and LMR (B,C,D,E) via X-tile analysis. [file Image_1.TIF]
